# Different underlying aetiologies in patients presenting with ventricular tachycardia fulfilling task force criteria for arrhythmogenic right ventricular cardiomyopathy: initial suspicion based on the 12-lead electrocardiogram

**DOI:** 10.1093/europace/euaf136

**Published:** 2025-08-20

**Authors:** Jarieke C Hoogendoorn, Laurens P Bosman, Jeroen F van der Heijden, Arthur A Wilde, Maarten P van den Berg, Sing-Chien Yap, J Peter van Tintelen, Dennis Dooijes, Anneline S J M Te Riele, Katja Zeppenfeld

**Affiliations:** Department of Cardiology, Willem Einthoven Center for Cardiac Arrhythmia Research and Management (WECAM), Leiden University Medical Center, Albinusdreef 2, 2333 ZA Leiden, The Netherlands; Department of Cardiology, Division Heart and Lungs, University Medical Center Utrecht, University of Utrecht, Utrecht, The Netherlands; Department of Cardiology, Division Heart and Lungs, University Medical Center Utrecht, University of Utrecht, Utrecht, The Netherlands; Department of Cardiology, Haga Ziekenhuis, Den Haag, The Netherlands; Department of Cardiology, Heart Centre, Amsterdam UMC Location University of Amsterdam, Meibergdreef 9, Amsterdam, The Netherlands; Amsterdam Cardiovascular Sciences, Heart Failure and Arrhythmias, Amsterdam, The Netherlands; European Reference Network for Rare, Low Prevalence and Complex Diseases of the Heart (ERN GUARD-Heart ); Department of Cardiology, University Medical Center Groningen, University of Groningen, Groningen, The Netherlands; European Reference Network for Rare, Low Prevalence and Complex Diseases of the Heart (ERN GUARD-Heart ); Department of Cardiology, Thorax Center, Cardiovascular Institute, Erasmus Medical Center, Rotterdam, The Netherlands; Department of Genetics, University Medical Center Utrecht, University of Utrecht, Utrecht, The Netherlands; Department of Genetics, University Medical Center Utrecht, University of Utrecht, Utrecht, The Netherlands; Department of Cardiology, Division Heart and Lungs, University Medical Center Utrecht, University of Utrecht, Utrecht, The Netherlands; European Reference Network for Rare, Low Prevalence and Complex Diseases of the Heart (ERN GUARD-Heart ); Department of Cardiology, Willem Einthoven Center for Cardiac Arrhythmia Research and Management (WECAM), Leiden University Medical Center, Albinusdreef 2, 2333 ZA Leiden, The Netherlands

**Keywords:** Arrhythmogenic right ventricular cardiomyopathy, Cardiac sarcoidosis, Ventricular tachycardia, Electrocardiogram

## Abstract

**Aims:**

The task force criteria (TFC) for arrhythmogenic right ventricular cardiomyopathy (ARVC) are highly sensitive but lack specificity. Atypical RV involvement (aRVi) may indicate different underlying aetiologies and prognosis, requiring specific therapeutic interventions. We aimed to evaluate the role of the baseline 12-lead ECG for initial suspicion of aRVi.

**Methods:**

From the Netherlands Heart Institute Arrhythmogenic Cardiomyopathy (NHI-ACM) registry, patients were selected who (i) fulfilled TFC for definite ARVC, (ii) presented with sustained ventricular tachycardia (VT), and (iii) underwent genetic testing. The first available ECG after VT was evaluated. PR prolongation ≥220 ms and/or a surface area of the maximum R′-wave in V1–V3 of ≥1.65 mm^2^ was defined as an aRVi-ECG. Patients with an ARVC-related pathogenic/likely pathogenic variant (P/LP+) were classified as ‘ARVC’. Data of P/LP− were reviewed by an expert panel and classified as either ‘ARVC’ or ‘different aetiology’ based on consensus.

**Results:**

A total of 159 patients were included (122 P/LP+ and 37 P/LP− patients). Nineteen patients had an aRVi-ECG [11 (9%) P/LP+ vs. 8 (22%) P/LP−, *P* = 0.038]. Of the P/LP− patients, 17 (46%) were classified as ‘different aetiology’ (e.g. myocarditis, ischaemia, sarcoidosis), including all 8 patients with an aRVi-ECG. Among the P/LP+ patients with an aRVi-ECG, 46% carried the p.Arg14del phospholamban pathogenic variant, and 64% died compared to 15 and 19% of P/LP+ patients without an aRVi-ECG, respectively (both *P* < 0.01).

**Conclusion:**

In P/LP− patients presenting with VT and fulfilling TFC, an aRVi-ECG should raise suspicion for a different underlying aetiology. In P/LP+ patients, an aRVi-ECG may identify those with poor outcome.

## Graphical Abstract

**Graphical Abstract:**
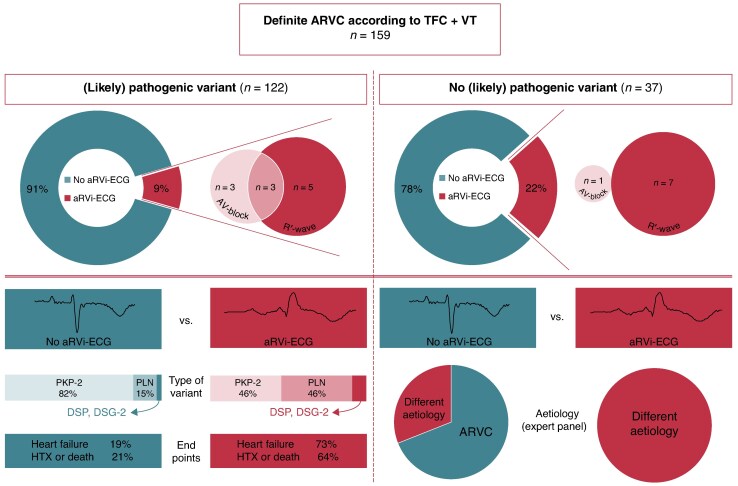
ARVC, arrythmogenic right ventricular cardiomyopathy; aRVi-ECG, atypical right ventricular involvement electrocardiogram; DSG-2, desmoglein-2; DSP, desmoplakin; HTX, heart transplantation; PKP-2, plakophilin-2; PLN, phospholamban; TFC, task force criteria; VT, ventricular tachycardia

What's new?

- Almost half of the patients fulfilling task force criteria, but without a pathogenic or likely pathogenic variant (P/LP−) may have a different aetiology during follow-up.
- The presence of an aRVi-ECG (PR prolongation and/or a surface area of the maximum R′-wave in V1–V3 of ≥1.65 mm^2^) in P/LP− patients should raise the suspicion for a different aetiology and should prompt adequate additional testing.
- In patients with a pathogenic or likely pathogenic variant (P/LP+), an aRVi-ECG may be indicative for a poor outcome, warranting closer follow-up in those patients.

## Introduction

Arrhythmogenic right ventricular cardiomyopathy (ARVC) is a hereditable cardiomyopathy leading to myocardial fibrofatty replacement, typically involving the right ventricle (RV).^1^ The diagnosis is established using the revised 2010 Task Force Criteria (TFC).^2^ Although in the majority of patients an ARVC-related likely pathogenic or pathogenic variant (P/LP+) can be identified, in approximately one-third of the patients, no associated likely pathogenic or pathogenic variant (P/LP−) can be found.^3^ The differential diagnosis in P/LP− patients presenting with ventricular tachycardias (VTs) of presumed RV origin includes (post-)myocarditis, cardiac sarcoidosis, (inherited) dilated or non-dilated left ventricular cardiomyopathy with septal or RV involvement, prior ischaemia, or an ARVC phenotype due to a variant in a yet unknown gene.^4,5^ The TFC for the diagnosis of ARVC do not require a P/LP variant or histological fibrofatty replacement at biopsy, making the TFC non-specific for ARVC vs. other RV cardiomyopathies.^6^ Diagnosis of the underlying aetiology has, however, important implications for counselling, treatment, and prognostication for the patient and the families.^7,8^

Recently, we have developed and validated a 12-lead electrocardiogram (ECG) algorithm to distinguish right-sided cardiac sarcoidosis from ARVC in patients presenting with a ventricular tachycardia (VT) of RV origin. This algorithm includes PR prolongation and the presence of an R′-wave in V1–V3 with a surface area (SA) ≥ 1.65 mm^2^, reflecting septal involvement and a transmural RV scar distribution considered atypical for classical ARVC.^9^

The aim of the current study was to determine the prevalence of atypical RV involvement (aRVi) on the 12-lead ECG among patients fulfilling TFC for definite ARVC and to evaluate the value of the ECG algorithm to identify patients with a potential different underlying aetiology among P/LP− patients.

## Methods

### Study population

From the Netherlands Heart Institute Arrhythmogenic Cardiomyopathy (NHI-ACM) registry, patients were selected who (i) fulfilled TFC for definite ARVC, (ii) had a sustained VT event, (iii) underwent genetic testing, and (iv) had a non-paced ECG available after presentation with VT. A sustained VT event was defined as documented VT lasting >30 s, out-of-hospital cardiac arrest (OHCA), and/or appropriate implantable cardioverter defibrillator (ICD) intervention (both anti-tachypacing and/or shock).

The NHI-ACM registry is a national observational cohort study predominantly including patients fulfilling TFC and their relatives.^10^ It is registered at the Netherlands Trial Registry (NTR7097) and has been approved by the local Dutch ethical committees (Leiden, N19.050; Utrecht, 18–126/C; Rotterdam, MEC 2018-1540). The current study complies with the Declaration of Helsinki.

### Data collection

In all patients, the first available ECG (25 mm/s and 10 mm/mV) in the database after presentation with VT was obtained. In case of an abnormal ECG, the pattern needed to be present in subsequent ECGs in order to exclude temporary changes after an arrhythmia. In addition, all available information regarding baseline characteristics and follow-up was retrieved from the registry. For the baseline characteristics, imaging data obtained closest to the time of the ECG recording were used.

### Arrhythmogenic right ventricular cardiomyopathy-related (likely) pathogenic variants

Pathogenic or likely pathogenic variants of plakophilin-2 (*PKP2*), desmoplakin (*DSP*), desmoglein-2 (*DSG2*), desmocollin-2 (*DSC2*), junction plakoglobin (*JUP*), and transmembrane protein 43 (*TMEM43*) have strong evidence for ARVC diagnosis, whilst phospholamban (*PLN*) and desmin (*DES*) genes have moderate evidence. For the purpose of this study, all strong- and moderate-evidence genes were considered as ARVC-related variants (P/LP+).^11^ The P/LP− group included all remaining patients, including those with pathogenic or likely pathogenic variants not related to ARVC. Genetic variants were classified according to the American College of Medical Genetics and Genomics guidelines and reviewed by two co-authors (J.P.v.T. and D.D.).^12^

### Data analysis and definitions

**Figure 1:**
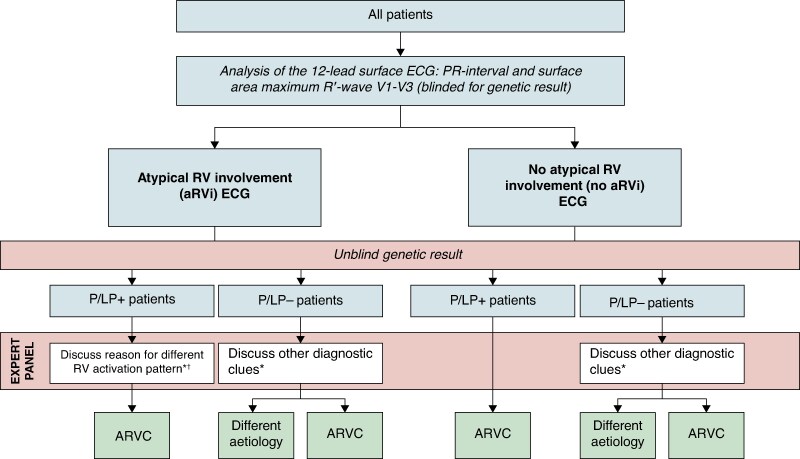
Study workflow. Study workflow as described in the Methods section. *These patients were discussed in an expert panel (see text). †Such as type of pathogenic variant and second aetiology. ARVC, arrhythmogenic right ventricular cardiomyopathy; ECG, electrocardiogram; P/LP+, ARVC-related pathogenic or likely pathogenic variant; P/LP−, no ARVC-related pathogenic or likely pathogenic variant; RV, right ventricle.

The study workflow is described in *Figure 1*. All ECGs were retrieved in pdf format and analysed blinded for all clinical data, including the results of genetic testing. Measurements were performed manually in Adobe Acrobat DC with 1200% zoom using the electronic measurement tool.

ECG parameters were collected and defined as previously described.^9^ The PR interval was determined in lead II or V5. A right bundle branch block (RBBB) was defined as QRS > 120 ms and (i) an R′ deflection in V1 or V2 and an S-wave of greater duration than R-wave in I and V6 or (ii) a pure dominant (notched) R-wave with an R-peak time >50 ms in V1 and normal R-peak time in V5 and V6.^13^ An atypical RBBB-pattern was defined as (i) an R′/S ratio <1 in V1^14^ or (ii) a QRS > 120 ms with R′ wave in V1 or V2, but without an S-wave of greater duration than R-wave in I and V6.

**Figure 2:**
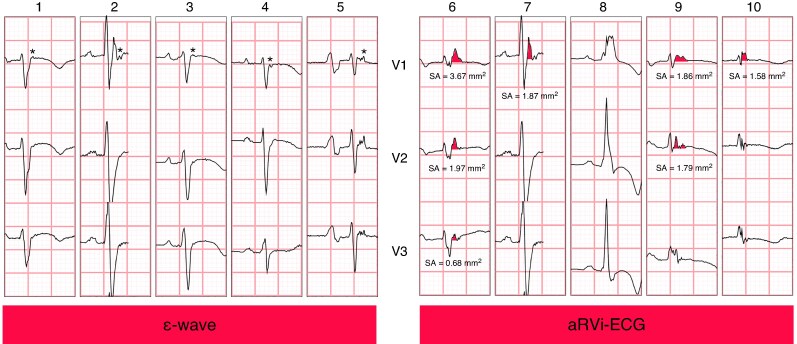
Examples of an epsilon wave and an aRVi-ECG. Examples showing the right precordial leads of five patients with an epsilon (Ɛ) wave on the left (marked with an asterisk) and of five patients with an atypical RV involvement electrocardiogram (aRVi-ECG) on the right. The measurement of the SA of the R′-wave in mm^2^ is shown when applicable. Please note, ECGs 2 and 7 are identical; this ECG fulfilled both criteria. 1: 38-year-old male with a laminopathy (*LMNA*-variant). 2/7: 38-year-old male with ARVC (homozygous *DSG2* variant). 3: 42-year-old male with ARVC (P/LP−). 4: 40-year-old female with ARVC (*PKP2* variant). 5: 69-year-old male with ARVC (*PKP2* variant). This ECG was also classified as aRVi-ECG with a SA of the maximum R′-wave (lead V2) of 1.95 mm^2^. 6: 58-year-old male with ARVC (*PLN* variant). 8: 35-year-old male with an inflammatory cardiomyopathy (P/LP−). 9: 42-year-old female with ARVC with progressive heart failure requiring transplantation (*PKP2* variant). 10: 20-year-old female with an inflammatory cardiomyopathy, most likely cardiac sarcoidosis (P/LP−). ARVC, arrhythmogenic right ventricular cardiomyopathy; aRVi-ECG, atypical right ventricular involvement electrocardiogram; ECG, electrocardiogram; SA, surface area.

An epsilon wave was defined as a low-amplitude signal distinct from the QRS complex in leads V1–V3, in at least three consecutive beats (*Figure 2*).^2^ An R′-wave was defined as any positive deflection immediately after an S-wave. When present, the SA of the R′-wave in V1–V3 was measured. A detailed description and examples of the measurement of the SA of the R′-wave are provided in the Supplementary material online, Methods and Supplementary material online, *Figure S1*. For this study, the SA of the R′-wave was measured by one observer (J.C.H.), considering the excellent agreement in SA between two observers in our prior study.^9^ Following these definitions, an epsilon wave and R′-wave may co-exist.

T-wave inversion (TWI) was defined in at least two contiguous inferior (II, III, and AVF) or lateral (I, AVL, V5, V6) leads. In addition, the major and minor repolarization criteria according to TFC were evaluated (TWI in V1–V3 in absence of RBBB; TWI in V1–V2 in absence of RBBB; TWI in V1–V4 in presence of RBBB; TWI in V4–V6).

### Evaluation of the electrocardiogram algorithm

AV conduction disturbances (PR ≥ 220 ms) and/or an SA of the maximum R′-wave in V1–V3 of ≥1.65 mm^2^ were classified as *positive* for aRVi based on ECG (aRVi-ECG). ECGs with an R′-wave with an atypical morphology (broad with or without additional fragmentation), which did not reach cut-offs for the SA of the R′-wave, were also classified as positive for aRVi (e.g. *Figure 2*, Case 10). Besides, a dominant R-wave was also classified as aRVi-ECG (e.g. *Figure 2*, Case 8). These ECGs were independently classified by two observers (J.C.H. and K.Z.).

### Expert panel

After analysis of the ECG, the genetic results were unblinded. Then, an independent expert panel including senior investigators from each including centre reviewed all available clinical data to determine the (likely) underlying aetiology of P/LP− patients. P/LP+ patients with an aRVi-ECG were also discussed in the expert panel to determine if other factors may explain or contribute to the aRVi pattern (e.g. type of pathogenic variant and second aetiology).

The expert panel discussed each patient in an online meeting based on all information available. Subsequently, patients were categorized as either ARVC or highly suggestive for a different aetiology. The latter is further referred to as ‘different aetiology’.

In the expert panel, previous reported distinguishing characteristics between ARVC and other RV cardiomyopathies were discussed. For example, an older age at first presentation and/or an atrioventricular block (during follow-up) and/or septal involvement on imaging or mapping and/or (biventricular) heart failure (HF) and related worse outcome [heart transplantation (HTX)/death] was considered less suggestive for ARVC.^6,15–17^ If possible, a likely or definite diagnosis was determined by the panel. If clinically relevant, additional testing [such as cardiac magnetic resonance (CMR), ^18^F-FDG positron emission tomography (PET), and biopsy] was advised by the panel.

### Statistical analysis

Statistical analysis was performed using IBM SPSS Version 29 (IBM Corporation, NY, USA). Categorical variables are expressed as numbers and percentages (%) and compared using *χ*² test or Fisher’s exact test. Continuous variables are expressed as mean ± SD or median (IQR) and compared between groups using Student’s *t*-test, Mann–Whitney *U* test, one-way ANOVA, or Kruskal–Wallis test when appropriate. A *P*-value of ≤0.05 was considered significant.

The diagnostic performance of an aRVi-ECG for identifying patients with a ‘different aetiology’ was determined using sensitivity, specificity, the positive predictive value (PPV), and negative predictive value (NPV). In addition, the Youden index was calculated (false-positive rate + false-negative rate − 1).^18^ The index ranges from 0 to 1, with 1 indicating a test with 100% sensitivity and specificity.

In P/LP+ patients, a comparison of the combined endpoint HF, HTX, or death in patients with and without an aRVi-ECG was performed using a Kaplan–Meier survival analysis, tested with the Mantel–Cox test.

## Results

### Study population

A total of 159 patients were identified from the database: 122 P/LP+ patients (mean age 42 ± 14 years, 66% male) and 37 P/LP− patients (mean age 43 ± 15 years, 92% male). Baseline characteristics are summarized in *Table 1*. The most common genetic variant involved the *PKP-2* gene (*n* = 96, 79%), followed by *PLN* (*n* = 22, 18%). Details on the nucleotide change and amino acid change are provided in Supplementary material online, *Table S1*. Patients with a P/LP− variants were more often male. However, age at presentation, RV structural abnormalities according to the TFC and LV involvement on echo and/or CMR were not significant different compared to P/LP+ patients. Only septal late gadolinium enhancement (LGE) on CMR was more often found in the group without a (likely) pathogenic variant (31 vs. 7%; *P* = 0.049).

**Table 1:** Baseline characteristics at time of ECG

	P/LP+ patients (*n* = 122)	P/LP− patients (*n* = 37)	*P*-value
Age, year	42 ± 14	43 ± 15	0.762
Male	81 (66)	34 (92)	**0.003**
Caucasian	119 (98)	37 (100)	1.000
Comorbidity			
Hypertension	5/76 (7)	4/24 (17)	0.212
Diabetes mellitus Type II	3/73 (4)	1/26 (4)	1.000
Hypercholesterolaemia	6/71 (9)	3/23 (13)	0.684
Myocardial infarction	1/74 (1)	2/26 (8)	0.165
Cerebrovascular accident	1/74 (1)	0/26 (0)	1.000
Sarcoidosis	2/74 (3)^a^	0/25 (0)	1.000
Age at first presentation, year	38 ± 14	41 ± 15	0.281
Reason of first presentation			0.166
Ventricular tachycardia	68 (56)	27 (73)	
Out-of-hospital cardiac arrest	10 (8)	2 (5)	
Syncope	4 (3)	2 (5)	
Palpitations and/or presyncope	27 (22)	3 (8)	
Abnormal test result	6 (5)	1 (3)	
Family screening	7 (6)	1 (3)	
Unknown	0 (0)	1 (3)	
Known with heart failure	4 (3)	1 (3)	1.000
Anti-arrhythmic drugs			
Class I	8 (7)	0 (0)	**0.047**
Class II	6 (5)	3 (8)	
Class III	49 (40)	8 (22)	
Class I and III	2 (3)	0 (0)	
None	47 (39)	24 (65)	
Unknown	10 (8)	2 (5)	
ICD	44 (36)	8 (22)	
Tissue available	44 (36)	18 (49)	0.283
TFC major	9 (21)	2 (11)	0.423
TFC minor	7 (16)	1 (7)	
Likely pathogenic or pathogenic variant			
Plakophilin-2 (*PKP2*)	96 (79)	NA	
Phospholamban (*PLN*)	22 (18)	NA	
Desmoglein-2 (*DSG2*)	2 (2)	NA	
Desmoplakin (*DSP*)	2 (2)	NA	
Other^b^	NA	1 (3)	
CMR available	86 (71)	30 (81)	0.216
Echo available	110 (90)	35 (95)	0.524
RV dilatation^c^	82/112 (73)	30/35 (86)	0.173
RV dysfunction^c^	63/108 (58)	19/33 (58)	1.000
RV hypokinesia^c^	20/109 (18)	5/34 (15)	0.797
RV dyskinesia, akinesia, or aneurysm^c^	60/109 (55)	18/34 (53)	0.846
LV dilatation^c^	15/115 (13)	7/35 (20)	0.412
LV dysfunction^c^	26/115 (23)	9/35 (26)	0.820
LV wall motion abnormalities^c^	20/115 (17)	9/35 (26)	0.329
Fatty infiltration on CMR	26/64 (41)	8/22 (36)	0.804
RV	25 (39)	8 (36)	1.000
LV	5 (8)	2 (9)	1.000
LGE-CMR performed	41/86 (48)	13/30 (43)	0.832
RV LGE	23/41 (56)	9/13 (69)	0.523
LV LGE	18/41 (44)	8/13 (62)	0.346
Septal LGE	3/41 (7)	4/13 (31)	**0.049**
Task force criteria imaging			
Major	51/115 (44)	15/34 (44)	0.152
Minor	8/115 (7)	6/34 (18)	

Numbers expressed as mean ± SD, median (IQR), or *n* (%). Bold values indicate *P*-values <0.05.

ECG, electrocardiogram; ICD, implantable cardioverter defibrillator; LGE, late gadolinium enhancement; LV, left ventricle; P/LP+, ARVC-related pathogenic or likely pathogenic variant; P/LP−, no ARVC-related pathogenic or likely pathogenic variant; RV, right ventricle; TFC, task force criteria.

^a^One patient (*PKP2*) with pulmonary sarcoidosis and one patient (*PKP2*) with biopsy-proven skin sarcoidosis.

^b^Namely, lamin A/C (*LMNA*).

^c^On echocardiogram or cardiac magnetic resonance (CMR).

### Electrocardiogram parameters in P/LP+ and P/LP− patients

*Table 2* lists the ECG parameters of P/LP+ and P/LP− patients. The ECG criteria included in the TFC [terminal activation duration (TAD) ≥ 55 ms, an epsilon wave or TWI] were not significantly different between groups. A typical complete right bundle branch block (CRBBB) was present in only one (1%) patient in the P/LP+ group, compared to four (11%) in the P/LP− group (*P* = 0.014). Regarding the parameters included in the ECG algorithm, PR prolongation did not differ between groups. However, the SA of the maximum R′-wave in V1–V3 was significantly larger in the P/LP− group compared to the P/LP+ group (median 0.43 vs. 0.17 mm^2^; *P* = 0.047).

**Table 2:** ECG parameters

	P/LP+ patients (*n* = 122)	P/LP− patients (*n* = 37)	*P*-value
Rhythm			
Sinus rhythm	118 (97)	37 (100)	0.574
Atrial paced	4 (3)	0 (0)	
Heart rate, b.p.m.	60 (53–74)	57 (52–70)	0.304
Conduction times			
PR ≥ 220 ms	7 (6)^a^	1 (3)	0.682
QRS > 120 ms	12 (10)	4 (11)	1.000
QTc, ms	422 ± 38	417 ± 24	0.420
Bundle branch block			
Typical CRBBB	1 (1)	4 (11)	**0.014**
Atypical CRBBB	5 (4)	0 (0)	
NIVCD	6 (5)	0 (0)	
TAD ≥ 55 ms^b^	52 (45)	16 (49)	0.843
Epsilon wave	11 (9)	2 (5)	0.734
T-wave inversion			
Inferior (II, III, AVF)	42 (35)	14 (38)	0.845
Lateral (I, AVL, V5, V6)	25 (21)	7 (19)	1.000
V1–V2 (minor)^b^	86 (75)	51 (64)	0.269
V1–V3 (major)^b^	77 (67)	18 (55)	0.219
V4–V6 (minor)	21 (17)	6 (16)	1.000
V1–V4 with CRBBB	4 (67)	4 (100)	0.467
None	17 (14)	5 (14)	1.000
Presence R′-wave V1–V3	46 (38)	13 (35)	0.847
V1	39 (32)	13 (35)	0.842
R′/S ratio V1	0.10 (0.05–0.34)	0.24 (0.08–1.75)	0.088
V2	17 (14)	4 (11)	0.785
V3	12 (10)	1 (3)	0.302
Maximum SA V1–V3, mm^2^	0.17 (0.10–0.48)	0.43 (0.15–2.40)	**0.047**
Maximum SA V1–V3 ≥ 1.65 mm^2^	6 (5)	3 (8)	0.435
aRVi-ECG	11 (9)	8 (22)	**0.038**

Numbers expressed as mean ± SD, median (IQR), or *n* (%). Bold values indicate *P*-values <0.05.

aRVi-ECG, atypical right ventricular involvement electrocardiogram; NIVCD, non-specific intraventricular conduction delay; P/LP+, ARVC-related pathogenic or likely pathogenic variant; P/LP−, no ARVC-related pathogenic or likely pathogenic variant; SA, surface area; TAD, terminal activation duration.

^a^One patient with PKP-2 variant had a PR of 274 ms during atrial pacing and sotalol 2 dd 160 mg and was therefore classified as no aRVi-ECG.

^b^In the absence of complete right bundle branch block (CRBBB).

### Presence of an atypical right ventricular involvement electrocardiogram

**Figure 3:**
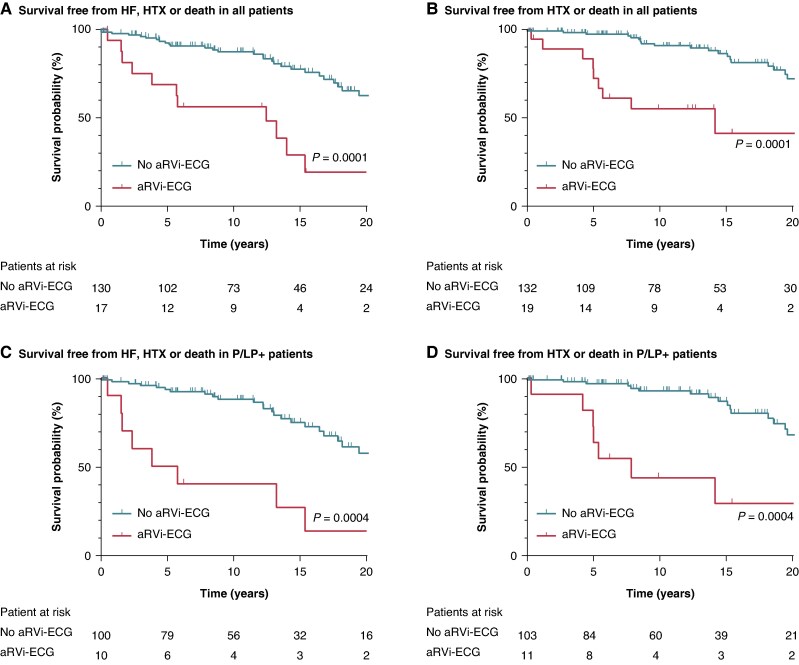
Combined endpoint in all patients and P/LP+ patients, stratified by aRVi-ECG at baseline. Survival probability, stratified by no atypical right ventricular involvement electrocardiogram (aRVi-ECG; green) and aRVi-ECG (red). (*A*) Free from heart failure (HF), heart transplantation (HTX), and death in all patients. (*B*) Free from HTX and death in all patients. (*C*) Free from HF, HTX, and death in patients with a pathogenic or likely pathogenic variant associated with ARVC (P/LP+). Note, four patients already had heart failure at the time of the ECG and were therefore excluded from this analysis. (*D*) Free from HTX and death in P/LP+ patients.

In 19 (12%) patients, an aRVi-ECG was present: 11 (9%) P/LP+ patients vs. 8 (22%) P/LP− patients (*P* = 0.038, *Graphical Abstract*). *Table 3* shows the comparison between patients with and without an aRVi-ECG in P/LP+ and P/LP− patients. The Kaplan–Meier curves for all patients regarding the combined endpoints stratified by an aRVi-ECG are provided in Figure *3A* and *B*.

**Table 3:** Comparison of patients with and without an aRVi-ECG in P/LP+ and P/LP− patients

	P/LP+ patients			P/LP− patients		
	No aRVi-ECG (*n* = 111)	aRVi-ECG (*n* = 11)	*P*-value	No aRVi-ECG (*n* = 29)	aRVi-ECG (*n* = 8)	*P*-value
Age	41 ± 14	55 ± 16	**0.002**	42 ± 15	47 ± 17	0.472
Male	76 (69)	5 (46)	0.179	27 (93)	7 (88)	0.530
Any LV involvement	30/104 (29)	6 (55)	0.096	10/28 (36)	5/7 (71)	0.112
LV dysfunction^a^	20/104 (19)	6 (55)	**0.016**	7/28 (25)	2/7 (29)	1.000
LV WMA^a^	16/104 (15)	4 (36)	0.098	6/28 (21)	3/7 (43)	0.340
LV LGE	15/37 (41)	3/4 (75)	0.303	4/9 (44)	4/4 (100)	0.105
Septal LGE	2/37 (5)	1/4 (25)	0.271	1/9 (11)	3/4 (75)	0.052
Type of P/LP variant						
*PKP2*	91 (82)	5 (46)^b^	**0.022**	NA	NA	
*PLN*	17 (15)	5 (46)		NA	NA	
*DSP*, *DSG2*	3 (3)	1 (9)^c^		NA	NA	
*LMNA*	NA	NA		1 (3)	0 (0)	1.000
Follow-up from the moment of ECG (years)	12 (5–18)	6 (4–15)	0.364	13 (7–21)	12 (2–14)	0.194
Heart failure	26 (24)	8 (73)	**0.002**	5 (18)	3 (38)	0.338
HTX or death	21 (19)	7 (64)	**0.003**	4 (14)	2 (25)	0.591
Expert opinion: ARVC	—	—		20 (69)	0 (0)	**0.001**

Bold values indicate *P*-values <0.05. ARVC, arrhythmogenic right ventricular cardiomyopathy; aRVi-ECG, atypical right ventricular involvement electrocardiogram; HTX, heart transplantation; LGE, late gadolinium enhancement; *LMNA*, lamin A/C; LV, left ventricle; *PKP2*, plakophilin-2; P/LP+, ARVC-related pathogenic or likely pathogenic variant; P/LP−, no ARVC-related pathogenic or likely pathogenic variant; *PLN*, phospholamban.

^a^By echo and/or CMR.

^b^Including two patients with a Class III variant in desmoplakin (DSP) or desmoglein-2 (DSG2).

^c^Homozygous *DSG2* variant.

P/LP+ patients with an aRVi-ECG showed more often LV dysfunction compared to those without an aRVi-ECG (55 vs. 19%; *P* = 0.016). Interestingly, patients with an aRVi-ECG showed a different distribution of the (likely) pathogenic variants: in patients with an aRVi-ECG, 46% carried the p.Arg14del *PLN* variant compared to only 15% of the no aRVi-ECG group (*P* = 0.01; *Graphical Abstract*). Moreover, two patients with a *PKP2* variant and an aRVi-ECG had a ‘second aetiology’, namely, ischaemic heart disease. Of note, two additional patients with a *PKP2* variant and an aRVi-ECG had a digenic variant, including a Class V PKP2 variant and a Class III variant in either *DSC2* or *DSP*.

In eight P/LP+ patients, data on follow-up were missing. During a median follow-up of 12 years (IQR: 6–19 years), 73% of patients with an aRVi-ECG at baseline developed HF, and 64% died or underwent HTX, compared to only 24 and 19% of the patients without an aRVi-ECG (*P* = 0.002 and 0.003, respectively). Of note, P/LP+ patients with an aRVi-ECG were significantly older compared to P/LP+ patients without an aRVi-ECG (*Table 3*). The worse outcome in P/LP+ patients with an aRVi-ECG remained significant when excluding patients with a *PLN* variant from analysis (*P* < 0.01). Figure *3C* and *D* shows the Kaplan–Meier curves for the combined endpoints in P/LP+ patients stratified by the presence of an aRVi-ECG at baseline.

In P/LP− patients with an aRVi-ECG, there was no significant difference in LV involvement, although patients with an aRVi-ECG tended to have more septal LGE on CMR compared to those without an aRVi-ECG (75 vs. 11%; *P* = 0.052). In P/LP− patients, the outcome was not significantly different, but patient numbers were small. Among the 8 patients with an aRVi-ECG, HF occurred in 3 (38%), and 2 (25%) underwent HTX or died, compared to 5 (18%) and 4 (14%) of the 27 patients without an aRVi-ECG (*P* = 0.338 and 0.591, respectively) (*Table 3* and Supplementary material online, *Figure S2*).

### Different aetiology

A total of 48 patients was discussed in the expert panel, including all P/LP− patients and the P/LP+ with an aRVi-ECG. The expert panel agreed that in 17 (46%) P/LP− patients, all available information was highly suggestive for a different aetiology. Differential diagnosis included cardiac sarcoidosis, myocarditis, ischaemic cardiomyopathy, and laminopathy. In 6/17 (35%) patients, the expert panel considered the potential importance of additional diagnostic tests (such as CMR, ^18^F-FDG PET, and biopsy), which would have been of help to further determine the definite underlying aetiology; 4/17 (24%) patients died, so no additional testing was suggested.

Importantly, all eight P/LP− patients with an aRVi-ECG were classified as ‘different aetiology’ by the expert panel, compared to nine (31%) of the P/LP− patients without an aRVi-ECG (*P* = 0.001; *Table 3*).

### Diagnostic performance of an atypical right ventricular involvement electrocardiogram for suspicion of a different aetiology

Among the total cohort, an aRVi-ECG at baseline had a 47% sensitivity and 92% specificity for identifying patients with a ‘different aetiology’ based on expert opinion (PPV, 42%; NPV, 94%). The Youden index was 0.39.

The most diagnostic challenging group is P/LP− patients. In this group, an aRVi-ECG had 47% sensitivity and 100% specificity, with a PPV of 100% and NPV of 69%. The Youden index in this group was 0.69.

## Discussion

This study investigated the role of an aRVi-ECG among a population fulfilling TFC and presenting with VT. It has several interesting findings. First, the prevalence of aRVi on the 12-lead ECG in this population was 12%. Furthermore, 46% of the P/LP− patients were classified as highly suggestive for a different aetiology by an expert panel, and all P/LP− patients with an aRVi-ECG at presentation with VT had a different aetiology. Also, P/LP+ patients with an aRVi-ECG more often carried the *PLN* p.Arg14del founder variant. Finally, among P/LP+ patients an aRVi-ECG was indicative for poor outcome.

### Typical right ventricular involvement in arrhythmogenic right ventricular cardiomyopathy and related electrocardiogram features

The pattern of RV involvement in ARVC has been described as subepicardial fibrofatty replacement, involving the RV inferior wall, anterior RVOT, and RV apex (the so-called triangle of dysplasia).^19^ Whilst the RVOT and peritricuspid inferior RV are affected early, RV apical involvement may be present only in very advanced stages of disease.^20–22^

This classical phenotype is reflected by typical ECG features. Delayed activation of the diseased peritricuspid area and RVOT may result in a TAD ≥55 ms and occasionally in a low-amplitude epsilon wave in the right precordial leads.^23–25^ The substrate for repolarization abnormalities (inverted T-waves) has not yet been fully elucidated, but it is thought to be caused by changes in the repolarization wavefront due to subepicardial fibrofatty replacement.^26^ These parameters are included in the current TFC and the presence of any ECG criterion has been reported in up to 90% of patients with ARVC.^4,27^

Considering the typical pattern of RV involvement in ARVC, both PR prolongation and an R′-wave with a considerable SA are atypical ECG features for classical ARVC.^9^ PR prolongation suggests septal involvement, which is rare in ARVC.^21,28^ A large R′-wave surface in V1–V3 requires late activation of a preserved peritricuspid RV and RVOT. These areas, however, show typically low voltages in classical ARVC, not significantly contributing to the surface ECG, giving occasionally rise to an epsilon wave, or are only detectable on a signal average ECG.^24,28^ Accordingly, PR prolongation and an SA of the maximum R′-wave of ≥1.65 mm^2^ were considered suggestive for aRVi (aRVi-ECG) in our study. Indeed, 91% of P/LP+ patients showed no aRVi-ECG.

### Atypical right ventricular involvement electrocardiogram in P/LP+ patients

Among the small proportion of P/LP+ patients with an aRVi-ECG, the type of (likely) pathogenic variant was different showing more patients carrying the *PLN* founder variant. In P/LP+ patients, an aRVi-ECG was indicative for a poor outcome, including HF, HTX, or death. This is in line with prior studies, showing AV conduction abnormalities in patients with a *PLN* variant.^29^ Furthermore, it is known that left ventricular dysfunction and HF is more than three times more common in patients with a *PLN* variant.^22,30^ This underlines the growing evidence of different phenotypes among different genetic variants associated with ARVC: ARVC with a *PLN* or *DES* variant may have a different phenotype compared to ‘typical ARVC’ with a *PKP2* variant.

### Different aetiology in patients fulfilling task force criteria

In the current study, almost half of the P/LP− patients had an aetiology other than ARVC as determined by an expert panel. Patients were included from an ARVC database which has collected patient data over time from academic hospitals with a special interest in non-ischaemic cardiomyopathies. It is possible that in less experienced centres, the proportion of patients with a different aetiology among those fulfilling TFC could be even higher. Although ARVC (fibrofatty replacement) is the most common aetiology in patients with VTs and an RV cardiomyopathy, there is a substantial risk of misdiagnosis.^27^ This is mainly due to the need of the TFC to establish the diagnosis. Of note, TFC have been validated against healthy subjects, and may not be discriminative for phenocopies of ARVC, such as myocarditis, cardiac sarcoidosis, other forms of (inherited) dilated cardiomyopathy, and RV infarction.^2,4,6,27^

However, it is of paramount importance to differentiate among the specific underlying aetiologies as this may guide specific treatment. For example, in ARVC and myocarditis, epicardial ablation is often needed, whilst in ischaemic heart disease endocardial ablation may be sufficient.^31^ Diagnosing active cardiac sarcoidosis and early initiation of immunosuppressive therapy may prevent deterioration of cardiac function.^7^ Also, indication for primary prevention ICD implantation differs among different aetiologies, some following specific risk calculators.^31^ Last, establishing the underlying disease has important consequences for family screening and follow-up.^8,31^

Of importance, scar patterns and scar distribution in phenocopies may be different, leading to a distinct RV activation pattern reflected by the surface ECG. Indeed, in our cohort all P/LP− patients with a different aetiology had an aRVi-ECG at presentation with VT. The presence of an aRVi-ECG had a 100% PPV and 69% NPV for a different aetiology in these patients. Based on our data, an aRVi-ECG should prompt additional testing, such as LGE-CMR, ^18^F-FDG PET, and/or biopsy, especially in P/LP− patients.

### Clinical implications and further perspectives

In P/LP− patients with an aRVi-ECG additional diagnostic studies, and in some patients, repeated testing should be considered to not miss alternative diagnoses. Given the high proportion of a different aetiology among P/LP− patients, regular re-evaluation should be performed, even in the absence of an aRVi-ECG.

In P/LP+ patients, an aRVi-ECG is associated with a poor outcome, warranting close follow-up. Further studies are needed to clarify whether patients with different pathogenic variants require specific treatment strategies and follow-up.^32^

### Limitations

In this study, an ECG algorithm was used which was originally developed and validated to distinguish between ARVC and right-sided cardiac sarcoidosis. This study shows that the presence of an aRVi-ECG may also be associated with other RV cardiomyopathies. Besides, we did not discuss all patients in an expert panel, and therefore among P/LP+ patients without an aRVi-ECG, there may be patients with a ‘second aetiology’, not suspected based on the 12-lead surface ECG.

Another limitation of this study is the classification of ECGs with ‘an R′-wave with an atypical morphology’ as aRVi-ECG. Any morphological criterion introduces subjectivity, like the epsilon wave. However, although the SA provides an objective criterion, there are drawbacks of one cut-off, considering a potential progressive myocardial disease with subsequent lower QRS amplitudes but also individual variations in QRS amplitudes due to extracardiac factors.

Due to the inclusion of patients from a national database which has been reviewed by experts in the field, the amount of patients with an aRVi-ECG is small, and hence the results should be interpreted with caution. However, data are retrieved from the largest European multicentre database, and it is unlikely that larger cohorts will be available in the near future. It is possible that an aRVi-ECG may also be indicative for a poor outcome among P/LP− patients. However, it did not reach statistical significance in this study likely due to the small patient numbers. Also, P/LP+ patients with an aRVi-ECG were significantly older (compared to P/LP+ patients without an aRVi-ECG), and this may affect the poorer outcome in this group. Larger (longitudinal) cohorts are required to evaluate the effect of age.

Due to the retrospective design using an existing database, we could not establish a definitive alternative diagnosis in patients with a suggested different aetiology. Further prospective studies are required to determine the definitive aetiology in these patients, performing and reporting the results of additional testing.

## Conclusion

An aRVi-ECG reflects a pattern of RV involvement not typical for classical ARVC. The presence of an aRVi-ECG should raise suspicion for a different aetiology in patients fulfilling TFC without a P/LP variant, and adequate additional testing should be initiated. In P/LP+ patients, an aRVi-ECG may identify patients with a higher likelihood of rapid disease progression.

## Supplementary Material

euaf136_Supplementary_Data

## Data Availability

The data underlying this article will be shared on reasonable request to the corresponding author.
