# Supplementary material for: Different underlying aetiologies in patients presenting with ventricular tachycardia fulfilling task force criteria for arrhythmogenic right ventricular cardiomyopathy: initial suspicion based on the 12-lead electrocardiogram
Source: Europace. 2025 Aug 20;27(8):euaf136. doi: 10.1093/europace/euaf136 (PMC12365759; doi:10.1093/europace/euaf136)
Supplement: euaf136_Supplementary_Data [file euaf136_supplementary_data.docx]

**SUPPLEMENTAL METHODS**

*Measurement of the surface area of the maximum R’-wave*

The measurement of the surface area of the maximum R’-wave was performed as previously described. ^1^ All measurements were performed using the measurement tool in Adobe Acrobat Pro DC with 1200% zoom. An R’-wave was defined as any positive deflection after an S-wave (and therefore a potential epsilon wave may also be measured). The surface area (SA) of the R’-wave was measured in three consecutive beats and subsequently averaged per lead. Examples are provided in **Supplemental Figure S1**.

**Supplemental Table 1: nucleotide change and amino acid change in patients with pathogenic or likely pathogenic variant**

|  | **Gene** | **Nucleotide change** | **Amino acid change** | **Number of patients** |
| --- | --- | --- | --- | --- |
| **ARVC-related** | **PKP2** | Deletion exon 1-4 | NA; deletion | 1 |
|  |  | Deletion exon 1-14 | NA; deletion | 3 |
|  |  | Deletion exon 7-14 | NA; deletion | 1 |
|  |  | Deletion exon 8 | NA; deletion | 1 |
|  |  | Deletion exon 10 | NA; deletion | 2 |
|  |  | c.148_151delACAG | p.Thr50Serfs*61 | 1 |
|  |  | c.258T>G | p.Tyr86* | 1 |
|  |  | c.235C>T | p.Arg79* | 13 |
|  |  | c.397C>T | p.Gln133* | 11 |
|  |  | c.917_918delCC | p.Pro318Glnfs*29 | 3 |
|  |  | c.1211dupT | p.Val406Serfs*4 | 11 |
|  |  | c.1212dupT | p.Leu404fs | 1 |
|  |  | c.1369_1372delCAAA | p.Gln457* | 2 |
|  |  | c.1378G>A | p.Asp460Asn | 1 |
|  |  | c.1748_1750delTTGinsAAT | p.Ile583_Asp585delinsLysTyr | 1 |
|  |  | c.1844C>T | p.Ser615Phe | 1 |
|  |  | c.1848C>A | p.Tyr616* | 4 |
|  |  | c.2014-1G>C | NA; Splice site | 1 |
|  |  | c.2028G>A | p.Trp676* | 1 |
|  |  | c.2062T>C | p.Ser688Pro | 1 |
|  |  | c.2146-1G>C | NA; Splice site | 7 |
|  |  | c.2203C>T | p.Arg735* | 1 |
|  |  | c.2386T>C | p.Cys796Arg | 17 |
|  |  | c.2489+1G>A | NA; Splice site | 4 |
|  |  | c.2489+4A>C | NA; Splice site | 3 |
|  |  | c.2509delA | p.Ser837Valfs*94 | 1 |
|  |  | c.2544G>A | p.Trp848* | 2 |
|  | **PLN** | c.40_42delAGA | p.Arg14del | 22 |
|  | **DSG-2** | c.137G>A | p.Arg46Gln | 1 |
|  |  | c.1003A>G (homozyg) | p.Thr335Ala | 1 |
|  | **DSP** | c.1060_1066delCT | Unknown | 1 |
|  |  | c.5419C>T | p.Gln1807* | 1 |
| **Non-ARVC-related** | **Lamin A/C** | c.1003C>T | p.Arg335Trp | 1 |

NA; not applicable.

**Supplemental Figure S1: Examples of the measurement of the surface area of the R’-wave
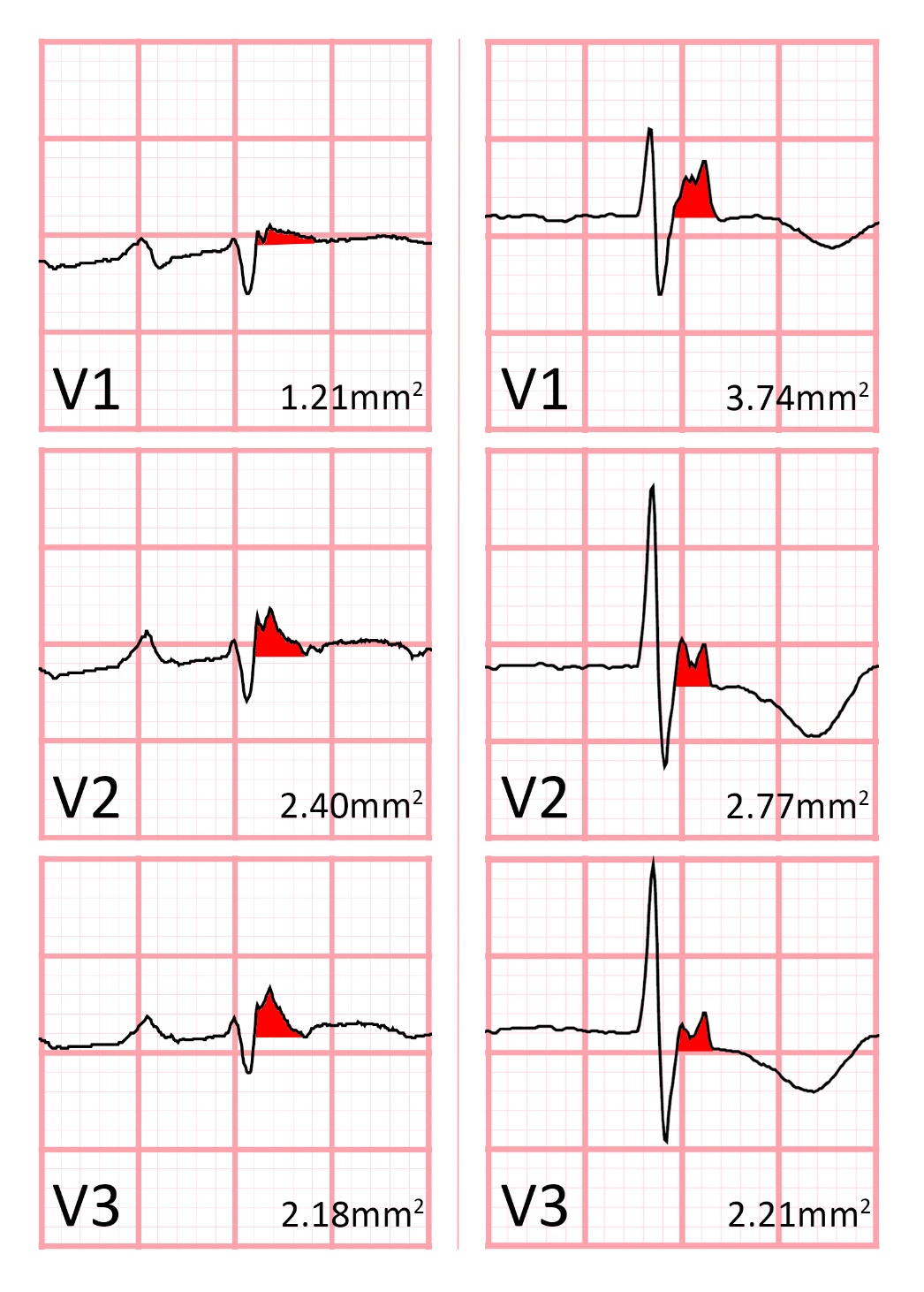
**

Examples of the measurement the surface area (SA) of the R’-wave in lead V1-V3 in two patients. The red area indicates the SA, the absolute value in mm^2^ is given for each lead.


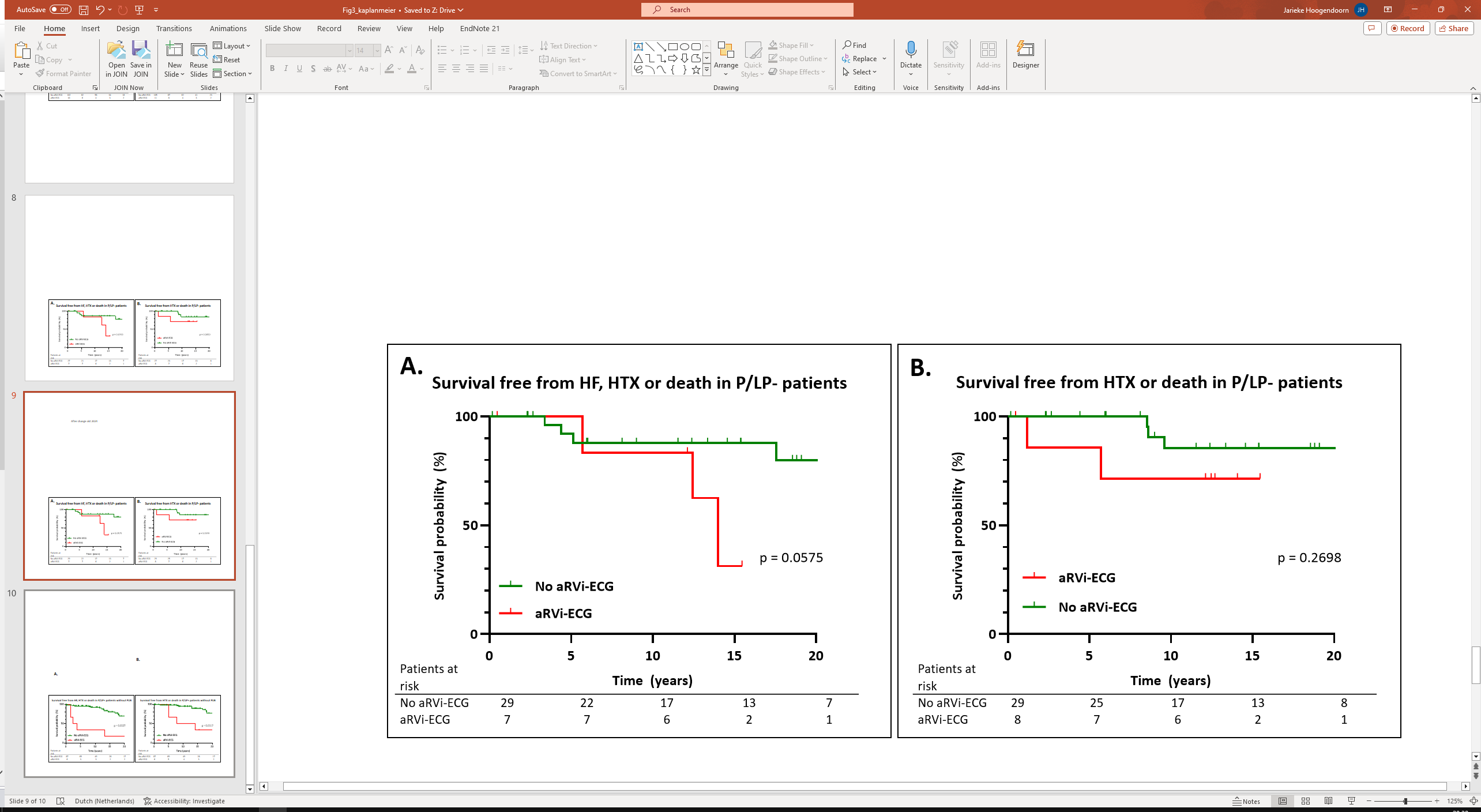
**Supplemental Figure S2: combined endpoint in P/LP- stratified by aRVi-ECG at baseline**

Survival probability, stratified by no atypical right ventricular involvement electrocardiogram (aRVi-ECG; green) and aRVi-ECG (red). A. Free from heart failure (HF), heart transplantation (HTX) and death. Note, 1 patient had already heart failure at the time of the ECG and was therefore excluded from this analysis. B. Free from HTX and death.

**SUPPLEMENTAL REFERENCES**1. Hoogendoorn JC, Venlet J, Out YNJ, Man S, Kumar S, Sramko M, et al. The precordial R' wave: A novel discriminator between cardiac sarcoidosis and arrhythmogenic right ventricular cardiomyopathy in patients presenting with ventricular tachycardia. Heart Rhythm. 2021.
